# Comparing meat abstainers with avid meat eaters and committed meat reducers

**DOI:** 10.3389/fnut.2022.1016858

**Published:** 2022-11-10

**Authors:** Muriel C. D. Verain, Hans Dagevos

**Affiliations:** Wageningen Economic Research, Wageningen University & Research, Wageningen, Netherlands

**Keywords:** meat abstainers, vegetarians, vegans, flexitarians, consumer segments, meat consumption curtailment, meat eaters, meat reducers

## Abstract

Shifting our eating patterns toward less animal-based and more plant-based diets is urgently needed to counter climate change, address public health issues, and protect animal welfare. Although most consumers agree that these are important topics, many consumers are not particularly willing to decrease the meat intensity of their diets. In supporting consumers to shift their diets, it is important to understand consumers’ attitudes, motivations, and preferences related to meat consumption and to take differences across consumers on these aspects into account. This study aims to in-depth research meat abstainers (vegetarians and vegans), and to explore how and to what extent they differ from avid meat eaters and committed meat reducers in terms of their (1) socio-demographic characteristics, (2) attitudes and norms, (3) food choice motives, and (4) food preferences and behavior. A survey has been conducted among a representative sample of Dutch adults. Comparisons show that meat abstainers (*N* = 198) differ from committed meat reducers (*N* = 171) and avid meat eaters (*N* = 344) on the four included categories of variables. In terms of demographics, we largely confirm the stereotype of vegans and vegetarians being highly educated females. In attitudes and norms, large differences exist with meat abstainers being least pro-meat and avid meat eaters being most pro-meat. Food choice motives confirm this, with meat abstainers valuing animal welfare and a good feeling higher than committed meat reducers and avid meat eaters. Finally, differences across the groups are most pronounced in terms of their food preferences and consumption, with a much higher appreciation of plant-based protein sources among meat abstainers, a high appreciation of non-meat animal-based proteins across committed meat reducers and a high appreciation of meat products among avid meat eaters. This shows that although differences across the groups are gradual and expected, in terms of reduction motivations and preferences of protein sources the three groups (frequent meat consumption-meat reduction-meat avoidance) are very distinct, which makes it unlikely to expect big shifts from one group to another in the short term.

## Introduction

More than a quarter of a century of scholarly attention has generated mounting scientific evidence about the pressing need for a dietary shift toward less animal-based and more plant-based diets in order to alleviate climate change, address public health issues and safeguard animal welfare. This field of research has made its way into such top-tier journals as *Science* (1, 2), *Nature* (3, 4), and *The Lancet* (5, 6). Despite broad scientific consensus on the urgency of shifting away from meat-heavy diets—first and foremost in high-income countries—in many western countries today’s consumption of (red and processed) meat is much higher than recommended (7), and on a global scale is meat consumption projected to rise in the upcoming decade (8, 9). Although meat-reduced (flexitarian) diets are slowly but gradually becoming more mainstream in various countries (10), and many consumers consider meat reduction as part of a healthy and sustainable diet (11), a large portion is not particularly willing to decrease their meat consumption (10, 12).

This also holds for the Netherlands, where a substantial increase in the number of self-identified flexitarians was observed in the past decade, but meat consumption remains relatively stable at a level beyond dietary recommendations (13, 14). Meat consumption patterns appear to be as notoriously difficult to change as other habitual behavior. Perhaps even more so, because of the strong symbolic meanings of meat, both socio-culturally (e.g., festivity, sense of belonging) and individually (e.g., strength, masculinity). Besides, various other reasons have been suggested to explain why people are “meathooked” (15) and attached to meat (16), ranging from liking the taste of meat and enjoyment of eating meat to limited cooking skills or culinary capital, as well as convenience, financial or family pressure factors. Simultaneously, however, also a small minority group exists today, with deep roots in Dutch food culture (17), who abstains from meat entirely. In spite of differences within and between (ovo-, lacto-, pesco-) vegetarians and vegans (18) meat abstainers have at least one main thing in common: they have meat cut out of their diet and can apparently resist the deeply-ingrained meat cravings of omnivores.

**FIGURE 1:**
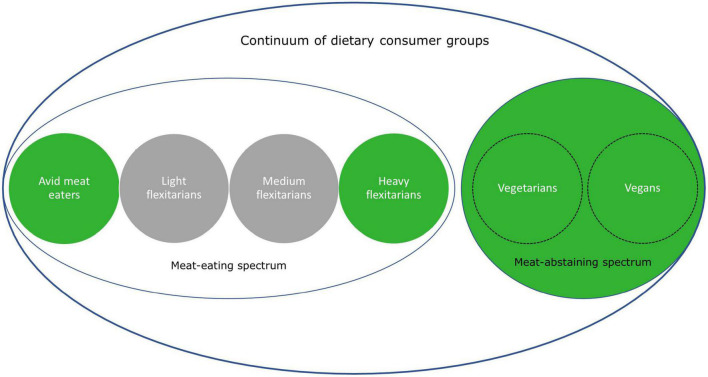
Continuum of dietary consumer group (green groups are included in the analysis).

Just for this reason a focus on comparing meat abstainers, who already made the dietary transition away from meat, with different dietary groups is of interest. Investigations into characteristics of meat abstainers shed light on how distinct this dietary consumer group is from full-time meat eaters at one side of the meat-eating spectrum and committed meat reducers at the other side (see Figure 1). These committed meat reducers—also known as semi-vegetarians or heavy flexitarians—are closest to meat abstainers in terms of their meat consumption but have not (yet) decided to completely abandon meat from their diet. Is this just an almost inconsiderable difference, or are flexitarians and vegetarians really distinct population subgroups? And if so, what characteristics differentiate these groups? Avid meat eaters in turn make completely different dietary choices than meat abstainers and committed meat reducers. Is their sumptuous meat consumption pattern reflected in their attitudes, motives and norms, or proof passionate meat eaters less distinct from meat abstainers than they seem to be? Getting more insightful answers to the questions how omnivores differ from meat abstainers improves our understanding of what to expect with respect to changing diets into less meat-centric directions. Some of our preliminary observations indicate that dietary shifts away from meat-rich diets appear anything but self-evident: flexitarianism is not necessarily a forerunner of vegetarianism (19, 20) and meat-reducing intentions have not resulted yet in a trend in which meat consumers move from light flexitarianism (mild reduction in meat consumption) toward more heavy flexitarianism (significant reduction in meat consumption) (14).

The present study follows a recent systematic review by Holler et al. (21) on differences between omnivores and vegetarians in which it was concluded that further studies about vegetarianism are needed—also in relation to adherents of meat-reduced diets. The current work also follows one of the suggestions for future research we have made in previous studies (10, 14), namely, to explore further how and to what extent meat abstainers differ from meat lovers and flexitarians.^1^ This study aims to in-depth research meat abstainers and compare them with avid meat eaters (i.e., self-declared meat eaters that consume meat every day of the week) and committed meat reducers (i.e., self-declared flexitarians that consume meat one or two times a week) on a broad range of characteristics: (1) socio-demographic features, (2) attitudes and norms, (3) motivational differences, and (4) food consumption preferences. By including a multitude of variables, we can provide a broad picture on similarities and differences across these three consumer groups.

The present study would also want to place itself in the research tradition devoted to commonalities and differences of vegetarianism in comparison to other—and more common—dietary forms of meat-attached consumers. This stream of literature originated a few decades ago (22, 23), and kept flowing thanks to studies like the ones by McEvoy et al. (24), Ruby (25), Rothgerber (26), De Backer and Hudders (27), De Backer and Hudders (28) or Allès et al. (29), and Mullee et al. (30). But particularly in recent years scholarly interest in comparing vegetarians, vegans, flexitarians, and omnivores has gained traction and turned into a blossoming field of study (21, 31–38). The goal of the current study is to add to this field of research by deepening our understanding in what characteristics particularly differentiate these dietary groups.

## Materials and methods

### Participants and procedure

Data was collected in an online survey in autumn 2019. Questions were posed in Dutch. A professional research agency (MSI-ACI Europe B.V.) recruited participants from existing panels by email. Quota were set for gender, age, and level of education, to get a balanced cross-sectional sample of the Dutch adult population. Vegetarians and vegans were oversampled, to retain a large enough group size for the analyses. Informed consent was organized at the level of the research agency and only anonymized data was shared with the researchers.

The initial dataset included 2,383 respondents. Data was cleaned by removing 203 participants who showed no dispersion in their answers, indicating insufficient effort. For a segmentation of all remaining meat consumers in the dataset we refer to Verain et al. (14). A subset of the data was used, since for this paper we were only interested in the poles of the continuum, i.e., those who do consume very limited amounts of meat or no meat at all vs. those who consume meat daily. This focus on the ends implies that a large middle segment of consumers who consume meat 3 to 6 days a week is neglected in the present study (see Figure 1). In total, 713 respondents were included in the analysis: 198 meat abstainers who self-identified as a vegetarian or vegan, 171 committed meat reducers who self-identified as a flexitarian/meat reducer and indicated to consume meat for diner 1 or 2 days a week, and 344 avid meat eaters who self-identified as a meat eater and indicated to consume meat for dinner 7 days a week (see below for the formulation of these questions).

### Measures

#### Socio-demographic characteristics

Screening questions were included on age (“what is your age?…”), gender (“I am a [man/woman]”) and level of education (“Could you indicate your highest level of education completed?”) (six answering options related to the Dutch educational system, recoded as low, middle and high, and an option “I’d rather not answer that” recoded as missing). In addition, questions were asked on household size (“How many people does your household consist of, including yourself?” and “How many of them are under 18”), household composition (“What is the composition of your household?” [Single without children (living at home)/Single with children living at home/Married/living together without children (living at home)/Married/living together with children living at home/Living with parents/Otherwise, namely…]), degree of urbanization of the residence (“What kind of place do you live in?” [In a village not adjacent to a city/In a village adjacent to a city/In a city of up to 30,000 inhabitants/In a city between 30,000 and 100,000 inhabitants/In a city of more than 100,000 inhabitants]) and country of birth of the respondent and its parents (“What is your country of birth?” “What is your father’s country of birth?” “What is your mother’s country of birth?” with answering options [The Netherlands/Turkey/Morocco/Suriname/Antilles/Aruba/Indonesia/Germany/Belgium/Poland/Other country]). Finally, participants were asked to *self-identify* as a meat eater, flexitarian, vegetarian, or vegan with the following question: “I would describe myself as a…[meat eater/meat reducer/flexitarian, I alternately eat meat and alternatives to meat/vegetarian, I do not eat meat/vegan, I do not eat or use any products of animal origin]”.

#### Attitudes and norms

Meat affection was measured with nine self-developed items based on the work by Lea and Worsley (39), Roos et al. (40), and Steptoe et al. (41). Factor analysis revealed two underlying constructs: appreciation of meatless meals and need for meat. *Appreciation of meatless meals* was measured with four items, with higher scores indicating a higher appreciation (Cronbach’s alpha α = 0.820). The included items were: “The day after a barbeque with meat, I eat less meat,” “I can do without meat for a day,” “I like a meal without meat,” and “It is easy to prepare a tasty meal without meat.” *Need for meat* was operationalized with five items (α = 0.876). The included items were: “After a day without meat I feel extra need for meat,” “Eating meat is an important part of who I am as a person,” “I think meat completes a meal,” “My family members or roommates like to eat meat,” and “If I do not eat meat for a whole day, I feel weaker.”

Ethical considerations concerning the consumption of meat were operationalized through seven items, adopted from de Boer et al. (42) and Vanhonacker and Verbeke (43) and were inspired by Lacroix and Gifford (44). Factor analysis revealed two underlying constructs. *Importance of environmental and animal welfare* was measured with three items (α = 0.860): “If I buy meat, I want to know it has been produced in an animal-friendly way,” “If I buy meat, I want to know it has been produced in an environmentally friendly way,” and “Animal wellbeing is important to me.” *Dislike of animals as a source for consumption* was measured with four items (α = 0.844): “The idea that meat comes from animals gives me an unhappy feeling,” “The consumption of meat is harmful to nature and the environment,” “Eating less meat is better for the environment,” and “I can accept that meat comes from animals.”

Three items dealt with the *price* of meat and were adopted from Steptoe et al. (41) and Eertmans et al. (45). Two items formed a reliable scale to measure whether meat was perceived as cheap (α = 0.844): “Meat is not expensive” and “Meat is too cheap.” One item dealt with value for money and is included as a single item.

*Perceived positive health effects* of reduced meat consumption were measured with five items, based on Lea and Worsley (39). After deleting one item (If I don’t eat meat, I don’t get enough nutrients) the scale was reliable (α = 0.899). The included items are: “Eating meat is unhealthy,” “Meat causes heart disease,” “Meat causes cancer,” and “Meat makes you fat.” The deleted item is included as a single item.

Three items were included that deal with *convenience* and ease of meatless meals, based on Malek et al. (46). Factor analysis revealed one construct to measure ease to prepare a meal without meat with two items (α = 0.960): “A meal without meat is easy to prepare” and “A meal without meat is easy to cook.” The other item is included as a single item and measures availability of meatless meals in shops.

*Personal norms* to consume less meat were operationalized with three items, based on Bamberg et al. (47) and Gärling et al. (48). One item was about the moral obligation to consume less/no meat and was asked in the same way to all respondents. This item is included as a single item. In addition, two items have been included that were adapted to the dietary group to which the respondent belongs. For meat consumers, the items measure their personal norm to consume less meat and for meat abstainers the items measure their personal norm to consume no meat. The included items were: “Because of my own values and norms, I feel morally obliged to eat [less/no] meat” and “It is important that people in general eat [less/no] meat” (α = 0.868).

Four items were included to measure *social injunctive norms* (α = 0.935). The items were based on Ajzen (49), Bamberg et al. (47), and Minton and Rose (50). The included items were: “People who are important to me think that I should eat less/no meat” and “I believe that my [friends/family/colleagues] want me to reduce/stop consuming meat.”

Perceived *status* of meat consumption was measured with four self-developed statements (α = 0.901), inspired by Roos et al. (40) and Twigg (51): “Eating meat is “cool,”” “By eating meat, I feel I am on top of the food chain,” “Eating meat gives one status,” and “By eating less meat I feel myself as being unworthy.”

Meat attachment was measured with two existing scales. The 16-item *Meat Attachment Questionnaire* (MAQ), developed by Graça et al. (16) was included. The four dimensions of the original MAQ-scale were computed by averaging the four items per dimension. All dimensions were reliable measures: hedonism (α = 0.953), affinity (α = 0.905), entitlement (α = 0.842), and dependence (α = 0.897). In addition, the 16-item *4Ns scale*, developed by Piazza et al. (52) was included. The original four dimensions were computed by averaging the four items per dimension. The Cronbach’s alpha value of the dimension “normal” is rather low, but the other dimensions were reliable measures: Natural (α = 0.863), Necessary (α = 0.922), Normal (α = 0.668), and Nice (α = 0.949).

All answers on the above-mentioned items were given on a seven-point Likert scale, ranging from “Totally disagree” (1) to “Totally agree” (7).

#### Food choice motives

Importance of 13 single-item food choice motives have been measured with the question “When purchasing food, I think the following characteristics are important…” The items were based on Onwezen et al. (53): “Healthy,” “animal friendly,” “safe,” “natural,” “convenient (preparation and purchase convenience),” “affordable,” “fairly produced (Fair Trade),” “sensory appealing (good taste, smell, and appearance),” “familiar to me,” “makes me feel good,” “environmentally friendly,” “from the region,” and “good for my waistline (weight).” The question has been repeated to ask for the motives that played a role in the decision to consume less or no meat. This question was only asked to the respondents who had indicated to have lowered their meat consumption in the past year or intend to do so in the coming year.

#### Food preferences and consumption

*Current meat consumption* was measured in average number of days per week a respondent consumes meat at the main meal, i.e., a warm meal at dinner. This question has not been asked to those who self-identified as a vegetarian or vegan. In addition, all respondents were asked about the number of days a week a person consumes a so-called *3-component meals* [a typical type of Dutch meals, consisting of three separate components for proteins, starch and vegetables, such as a sausage with potatoes and broccoli, comparable to the traditional “meat and two-three veg” dishes as mentioned by Kerslake et al. (54)], with or without meat and so-called *combined meals* (mixed ingredients, such as in a pasta dish, curry, or soup) with or without meat. Subsequently, the respondent was asked to select from a list of products what type of products he or she consumes when meat is left out of the dish (fish, plant-based meat substitutes, egg, cheese, tofu or tempeh, pulses, nuts, mushrooms, seaweed, insects, no alternative, or “other”). These questions have been based on Verain et al. (20).

Finally, the hierarchy of foods was used to measure *food preferences*. Respondents were asked for to rank a long list of protein sources, both animal-based and plant-based, from least preferred to most preferred [based on Twigg (51)]. The included products are displayed in Table 3.

### Analysis

Statistical analyses were conducted in SPSS (version 25.0). Exploratory factor analyses were conducted to form constructs of the items on attitudes and norms. Reliability was checked with Cronbach’s Alpha. Univariate analyses of variance (ANOVAs), with Games–Howell *post-hoc* comparisons of mean scores to test for significant differences between meat abstainers, committed meat reducers and avid meat lovers on the continuous variables. Cross-tabulations with Pearson chi-square tests were performed to test for significant differences between the dietary groups on categorical variables. Due to the unbalanced sample sizes and the violation of homogeneity of variance, the Brown–Forsythe and Welch F tests were conducted. Games-Howell *post-hoc* tests were performed because equal variances could not be assumed. This test is suitable when sample sizes are unequal, which is the case here [(55), p. 276].

## Results

### Socio-demographic characteristics

#### Characterization of meat abstainers

Meat abstainers are in majority female (79%), and this group has a mean age of 48 years. 14% has a lower level of education, 38% a medium level of education, and 48% has a high level of education. The average household size is 2.1 persons, and these are most frequently single households without kids at home (37%) or couples without kids at home (32%). Meat abstainers can be found in large cities (25%) as well as in rural villages (24%) and everything in between. 94% of meat abstainers in our sample are born in the Netherlands.

#### Comparing meat abstainers with committed meat reducers and avid meat eaters

The overrepresentation of females in the group of meat abstainers (79%) is similar among committed meat reducers (74%), but is in sharp contrast with avid meat eaters who are male in majority (65%). Meat abstainers are slightly younger than committed meat reducers [*F*_(2,710)_ = 3.193, *p* = 0.042] and are more often highly educated than avid meat eaters. Household size of meat abstainers is a bit larger than for committed meat reducers, but a bit smaller than for avid meat eaters [*F*_(2,710)_ = 13.621, *p* < 0.001]. Meat abstainers more often live in single households (37%) compared to avid meat eaters (Table 1).

**TABLE 1:** Socio-demographic characteristics per dietary consumer group.

	Meat abstainers	Committed meat reducers	Avid meat eaters
			
N	198	171	344
Male (%)	20.7^a^	25.7^a^	64.5^b^
Mean age (range)	48.0 (19–76)^a^	52.2 (18–81)^b^	49.2 (18–84)^a,b^
**Education level (%)**			
Low	13.6^a^	19.3^a^	21.2^a^
Middle	38.4^a^	36.8^a^	46.5^a^
High	48.0^a^	43.9^a^	32.0^b^
Household size	2.1^a^	1.8^b^	2.4^c^
**Household type (%)**			
Single	37.4^a^	47.4^a^	22.1^b^
Single with kids	8.1^a^	6.4^a^	5.5^a^
Partner	31.8^a,b^	28.1^a^	39.8^b^
Partner with kids	18.2^a,b^	13.5^a^	23.3^b^
Living with parents	4.5^a^	3.5^a^	7.8^a^
**Urbanization (%)**			
Rural village	23.7^a,b^	17.0^a^	27.9^b^
Village adjacent to a town	14.6^a^	14.6^a^	18.9^a^
Town < 30,000 inhabitants	13.1^a^	11.7^a^	9.9^a^
Town 30,000–1000,000 inhabitants	22.7^a^	20.5^a^	18.6^a^
City < 100,000 inhabitants	24.7^a,b^	35.1^a^	23.8^b^
**Origin**			
Born in NL (%)	94.4^a,b^	89.5^a^	96.5^b^
Father born in NL (%)	89.9^a,b^	85.4^a^	92.2^b^
Mother born in NL (%)	91.9^a,b^	84.2^a^	91.9^b^

^a–c^Different superscripts across rows indicate significant different means.

### Attitudes and norms

#### Characterization of meat abstainers

Meat abstainers do not derive status from consuming meat, have a low need for meat and are not attached to meat, indicated by their low scores on all dimensions of the Meat Attachment Questionnaire. In addition, they do not think that meat consumption is natural, necessary, normal, or nice. They highly appreciate meatless meals, believe that these are easy to prepare and well available in supermarkets. Meat abstainers score high on ethical considerations related to meat consumption such as animal welfare and environmental impact and they dislike the idea that meat comes from animals. They feel morally obliged to abstain from eating meat and have a high personal norm to avoid eating meat. In contrast, they do not perceive a high social norm to limit meat consumption. Meat abstainers believe that meat reduction can lead to some positive health effects, but this believe is not very strong and they do not see that a diet without meat would lead to deficiencies. Finally, they do not think that meat is expensive, but regardless they disagree that meat is worth the money (Table 2).

**TABLE 2:** Mean scores on meat-related attitudes and norms per dietary consumer group.

	Meat abstainers	Committed meat reducers	Avid meat eaters
Appreciation of meatless meal	6.49^a^	6.19^b^	3.28^c^
Need for meat consumption	1.62^a^	2.03^b^	5.05^c^
Importance of environment/animal friendliness	6.34^a^	5.53^b^	4.20^c^
Dislike of animals as source of meat	5.95^a^	4.56^b^	2.90^c^
Meat is not expensive	4.60^a^	3.70^b^	3.12^c^
Meat is worth its money	2.36^a^	3.88^b^	5.39^c^
Perceived positive health effects of less meat	4.58^a^	3.32^b^	2.31^c^
Deficiency without meat	1.74^a^	2.44^b^	4.51^c^
Convenience	6.51^a^	6.25^a^	4.34^b^
Availability	5.13^a^	5.35^a^	4.44^b^
Personal norm to consume less meat [meat consumer] or no meat [meat abstainers]	5.49^a^	4.97^b^	2.44^c^
Moral obligation to consume less/no meat	5.38^a^	4.32^b^	2.21^c^
Social norm	2.21^a,b^	2.28^a^	1.93^b^
Meat consumption gives status	1.35^a^	1.52^a^	2.68^b^
**Meat attachment questionnaire**			
Hedonism	1.38^a^	2.76^b^	5.76^c^
Affinity	2.88^a^	4.78^b^	5.99^c^
Entitlement	2.10^a^	2.87^b^	5.22^c^
Dependence	1.29^a^	2.03^b^	4.68^c^
**4N’s**			
Natural	2.10^a^	3.17^b^	5.15^c^
Necessary	1.65^a^	2.68^b^	4.93^c^
Normal	2.76^a^	3.17^b^	4.76^c^
Nice	1.41^a^	2.50^b^	5.52^c^

^a–c^Different superscripts across rows indicate significant different means.

#### Comparing meat abstainers with committed meat reducers and avid meat eaters

Meat abstainers significantly differ from avid meat eaters on all included variables, except for social norms (Table 2), where avid meat eaters unsurprisingly score more in favor of meat consumption and less in favor of meatless meals. Differences are particularly pronounced for need for meat, meat attachment and the believe that meat consumption is nice.

The difference between meat abstainers and committed meat reducers is much smaller, although also between these two groups almost all included variables differ significantly in the expected direction (except for status, convenience, availability, and social norms). These two groups differ the most in their affinity with meat, dislike of animals as source of meat, positive health effects of meat reduction and the believe that meat is worth its money. Overall, the groups are very distinct in their attitudes and norms, with two exceptions: meat consumption does not seem to give status in any of the groups and social norms to reduce meat consumption are perceived to be low in all groups (Table 2). Differences are most outspoken between avid meat eaters and the other two groups.

### Food choice motives

#### Characterization of meat abstainers

**FIGURE 2:**
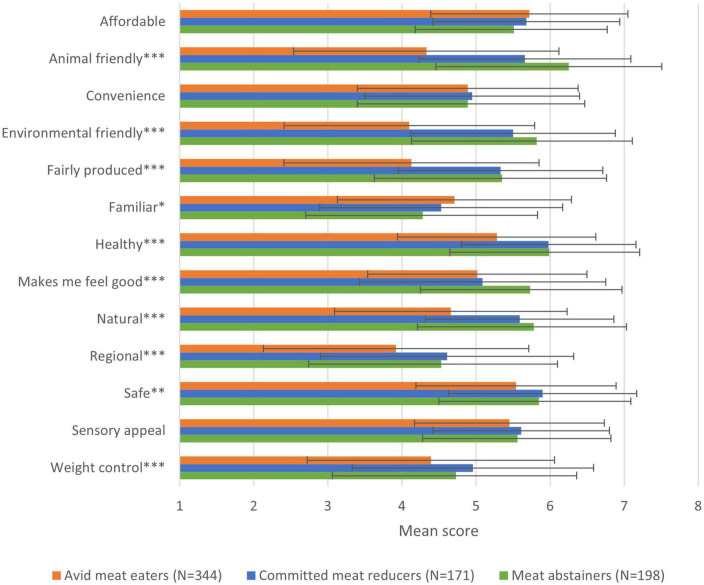
Mean scores of food choice motives per dietary consumer group (*p < 0.05, **p < 0.01, ***p < 0.001).

**FIGURE 3:**
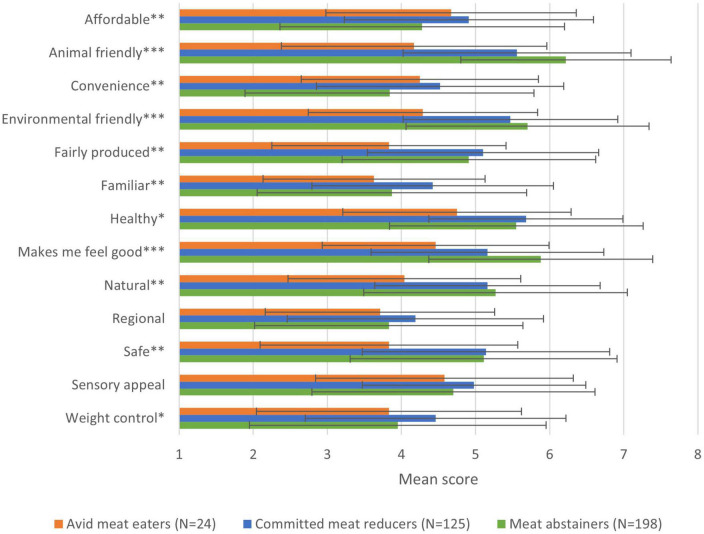
Mean scores of motives to limit meat consumption per dietary consumer group (*p < 0.05, **p < 0.01, ***p < 0.001).

Animal welfare is the most important motive for meat abstainers in selecting their food, followed by healthiness, food safety, environmental welfare, and naturalness. Regional and familiarity are least important to them, although the absolute scores indicate that all included food choice motives are important to meat abstainers (all scores above neutral) (Figure 2). Animal friendliness is also the most important motive for meat abstainers to have stopped eating meat. In addition, “makes me feel good,” environmental friendliness, health, and naturalness are important motives for stopping (Figure 3).

#### Comparing meat abstainers with committed meat reducers and avid meat eaters

Animal welfare, the most important food choice motive for meat abstainers, is not in the top three motives of committed meat reducers and avid meat eaters. For committed meat reducers, health is most important, followed by food safety and affordability. For avid meat eaters, affordability is most important, followed by food safety and sensory appeal. Like for meat abstainers, animal welfare is the number one motive for committed meat reducers to have reduced their meat consumption, followed by environmental friendliness and healthiness. The small number of avid meat eaters that has reduced indicate healthiness as the most important reason, followed by affordability and sensory appeal.

When comparing the mean scores, a lot of differences between the groups can be found in terms of their motivations. Animal welfare is significantly more important to meat abstainers than to the other two groups (and more important to reducers than to avid meat eaters). In addition, “makes me feel good” is more important to meat abstainers than to the other groups. On the other motives, meat abstainers do not differ from committed meat reducers, but the differences with avid meat eaters are almost all significant (except for sensory appeal, affordability, and convenience), with avid meat eaters attaching a higher importance to familiarity and a lower importance to all other motives (Figure 2).

Also in their motives to reduce, the groups differ much. Compared to committed reducers, animal friendliness and “makes me feel good” are more important motives to reduce for meat abstainers, whereas affordability, weight control, familiarity, and convenience are less important motives to meat abstainers. Compared to avid meat eaters, animal friendliness, “makes me feel good,” environmental friendliness, health, naturalness, food safety, and fair production are more important reasons to reduce for meat abstainers (Figure 3).

### Food preferences and consumption

#### Characterization of meat abstainers

Meat abstainers consume 3-component meals (three separate components for proteins, starch, and vegetables, such as a sausage with potatoes and broccoli) without meat about 4 days a week. When consuming a 3-component meal, most of the abstainers replace meat with plant-based meat substitutes, eggs, pulses, or mushrooms. And 45% does not replace meat by another product. Meat abstainers also consume combined meals without meat (mixed ingredients, such as in a pasta dish, curry, or soup) about 4 days a week. When consuming a combined meal, plant-based meat substitutes are also the most used alternatives, followed by pulses and mushrooms, but also egg, cheese, tofu, and tempeh and nuts are used as replacers by about half of the meat abstainers and 44% does not use any meat replacer in combined meals.

In terms of appreciation of different types of protein-rich products, meat abstainers value mushrooms, cashews and vegetarian burgers most, followed by Dutch cheese and chickpeas. Meat products are less liked as protein sources by this group.

#### Comparing meat abstainers with committed meat reducers and avid meat eaters

Committed meat reducers consume 3-component meals without meat with the same frequency as meat abstainers (on average 4 days a week). Avid meat eaters consume such type of meals much less often (less than 1 day a week). Combined meals without meat are a little less frequently consumed by committed meat reducers (3 days a week) compared to meat abstainers (4 days a week). Avid meat eaters consume such types of meals much less frequently (less than 1 day a week).

**FIGURE 4:**
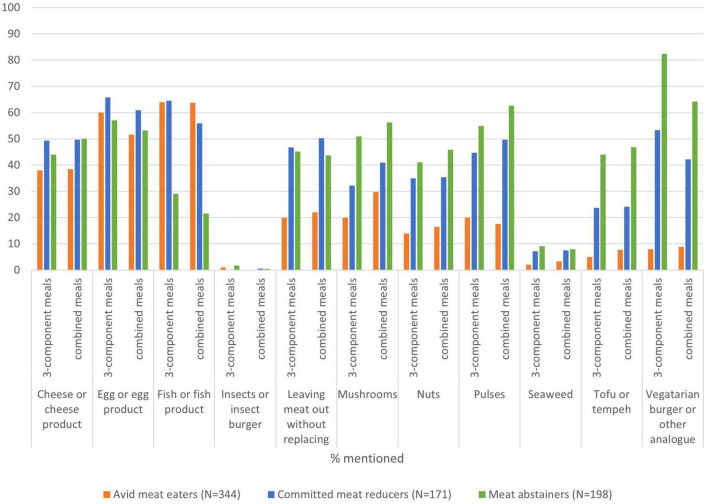
Meat replacers in 3-component meals and combined meals per dietary consumer group.

The groups also differ in the type of protein source they consume in meatless meals (see Figure 4). Plant-based meat substitutes are consumed by a much larger proportion of meat avoiders, whereas fish is much more frequently consumed by committed reducers and avid meat eaters. This holds for both types of meals. The differences between avid meat eaters and meat abstainers are particularly large in the consumption of plant-based meat substitutes in 3-component meals (8% as opposed to 80%). Eggs and cheese products are also regularly used to replace meat in both types of meals, by an equal proportion of consumers in each group. Mushrooms and tofu or tempeh are more frequently used by meat abstainers than by the other groups, and nuts are equally often used by meat abstainers and committed meat reducers, but less frequently by avid meat eaters.

The groups differ greatly in their appreciation of different types of protein-rich products. Whereas the top three preferred products of meat abstainers are all plant-based products (mushrooms, cashew, vegetarian burgers), the two most liked products of committed meat reducers are animal-based products other them meat (eggs and cheese) and the three favorite products of committed meat eaters are meat products (steak, chicken filet, and meat balls). The contrast in appreciation of plant-based products is striking, scoring in the top favorite products of meat abstainers and in the bottom part of avid meat eaters. For meat products, the opposite is true (Table 3).

**TABLE 3:** Ranking of meat products and meat alternatives from most liked to least liked per dietary consumer group.

Meat abstainers		Committed meat reducers		Avid meat eaters	
Mushrooms	6.0	Eggs	6.2	Steak	5.6
Cashews	6.2	Dutch cheese	6.3	Chicken filet	5.7
Vegetarian burgers	6.3	Mushrooms	7.8	Meat balls	6.3
Dutch cheese	7.0	Cashews	7.8	Eggs	7.2
Chickpeas	7.2	Salmon steak	8.4	Dutch cheese	7.7
Eggs	7.3	Peanuts	9.1	Chop	7.8
Vegetarian minced meat	7.4	Vegetarian burger	9.4	Hamburger	7.9
Kidney beans	7.5	Kidney beans	9.4	Fried fish	8.8
Peanuts	8.1	Chickpeas	9.9	Frikandel	9.2
Tofu	8.6	Chicken filet	10.3	Salmon steak	9.9
Shiitakes	9.1	Fried fish	10.7	Mushrooms	10.1
Seaweed burger	10.1	Vegetarian minced meat	11.0	Peanuts	10.7
Salmon steak	12.7	Meat balls	11.9	Cashews	10.8
Fried fish	13.7	Tofu	12.4	Kidney beans	11.5
Insect burger	15.0	Shiitakes		Chick peas	14.9
Chicken filet	15.5	Steak	12.8	Shiitakes	15.0
Meat balls	15.6	Hamburger	13.3	Vegetarian minced meat	15.7
Hamburger	16.0	Seaweed burger	14.4	Vegetarian burger	15.8
Frikandel	16.8	Frikandel	15.1	Tofu	16.2
Steak	16.9	Chop	15.3	Seaweed burger	16.7
Chop	17.8	Insect burger	16.9	Insect burger	17.4

## Discussion

### Main findings

#### Differences and similarities in socio-demographic characteristics

This is the first Dutch study that not only characterizes meat abstainers (vegetarians and vegans) but compares them with committed meat reducers (consuming meat 1 or 2 days a week) and avid meat eaters (consuming meat 7 days a week) on a broad spectrum of variables: socio-demographics, attitudes, and norms, motivations and food preferences and consumption. The study aimed to explore what characterizes meat abstainers, and whether they are really a distinct subgroup of the population—as is sometimes implicitly or explicitly suggested.

Based on a large national representative sample of the Dutch adult population we confirm the stereotype of vegans and vegetarians as being mainly highly-educated females. This outcome engages with the extant literature (29, 33, 56, 57). Meat abstainers and meat reducers are quite similar in terms of their socio-demographic characteristics, though committed reducers are on average a bit older and live in smaller households. The difference is bigger with avid meat eaters, as they are mainly males, less often highly educated, living in larger household and less often single. Interestingly, the stereotype may be even stronger for committed meat reducers as most differences in terms of demographics exist between committed meat reducers and avid meat eaters. These results are largely in line with findings in Australia by Malek and Umberger (33) on differences between the groups in gender, age, household type and education level. Recent findings from New Zealand by Kemper et al. (58) on differences between three dietary groups of meat eaters and meat reducers also showed that more meat-reducing consumers were more likely to be female and higher educated while meat eaters were more likely to be male and less educated. Please note, however, that prior research found that the explanatory value of socio-demographics in explaining (sustainable) food consumption is limited as opposed to for example psychographic factors (59).

#### Pro-meat attitudes and norms

In terms of their attitudes and norms, large differences exist between the three groups in the expected direction, with meat abstainers being least pro-meat and avid meat eaters being most pro-meat. As expected, meat abstainers are not attached to meat in any aspect, are positive about meatless meals, and dislike the idea that meat comes from animals. This may be explained by personality aspects such as their greater openness and empathy as compared to omnivores (21). Moreover, meat abstainers attach high importance to ethical aspects related to meat, such as animal welfare and environmental impact, which is in accordance with previous literature (35, 60). Committed meat reducers differ from meat abstainers with regard to their attitudes and norms toward meat reduction, although the differences are generally small. It seems that committed meat reducers have a more nuanced opinion on meat consumption compared to meat abstainers. The most pronounced difference is in terms of affinity with eating meat, with committed meat reducers expressing much less feelings of repulsion.

The difference with avid meat eaters is much larger as they are much more attached to meat, are more positive about meat consumption, and favor meatless meals less. They attach less importance to ethical considerations and have no problem with animals being a source of consumption, possibly explained by their higher orientation toward social dominance (21). Interestingly, social norms are perceived to be low in all groups, and do not differ between meat abstainers and the other two groups. This is in line the work by Müssig et al. (35), who found that social norms were among the least important eating motives for both omnivores and meat abstainers.

#### Motives to reduce

Regarding their food choice motives, differences are again in the expected direction but provide some interesting insights. To meat abstainers, the most important food choice motives are motives related to sustainability and health aspects: animal welfare, healthiness, food safety, environmental welfare, and naturalness. Studies by Malek and Umberger (33) and North et al. (36) confirm that animal welfare is the most important motive for meat abstainers, and more specific for vegans (61), but contrary to our results, Malek and Umberger (33) show that for all dietary groups, price, and taste are among the five most important motives. Kemper et al. (58) found that taste is a main reason for meat consumption while price is an important motive for meat reduction. Price and taste are often mentioned as prerequisites to all population subgroups, but this is not reflected in our study. It might have to do with the trend that sustainability and health aspects are becoming more and more valued by consumers (62), and might start to dominate other egocentric motives, at least in certain subgroups. In comparing the dietary consumer groups, we found that the absolute scores on the food choice motives of meat abstainers do not differ from committed meat reducers, except for animal friendliness and “makes me feel good.” This is in contrast with the findings by Malek and Umberger (33) that motives of unrestricted omnivores are similar to those of meat reducers but differ from those of vegetarians and vegans. This contrast with our study may have to do with the less strict definition of meat reducers in their sample. The finding that animal friendliness is discriminating between meat abstainers and the other groups is in line with other studies (27, 28, 33, 35, 63, 64). Animal welfare is not only the most important food choice motive to meat abstainers but is also the most important motive for them to have stopped consuming meat, followed by “makes me feel good,” environmental welfare, health reasons and naturalness. This is mostly in agreement with previous studies that identify environmental, social, animal welfare, and health concerns as the main motivators of meat reduction (31, 35, 65, 66).

The finding that “makes me feel good” is the second most important reason to meat abstainers to have stopped eating meat is surprising. Müssig et al. (35) for example found that affect regulation (…to feel good) ranks in the bottom part of food choice motives. The high score on “feeling good” in our study may be explained by a recent qualitative research by Simons et al. (67), who found that meat avoidance is, next to ethical and health motivations, also unconsciously driven by aspects such as empowerment, enrichment, autonomy, and superiority that make one feel good. This feeling-good is less pronounced for committed meat reducers. Surprisingly, the top three of most important motives to reduce meat consumption for committed meat reducers are all egoistic motives: health, food safety, and affordability. This also holds for avid meat eaters. In general, this group attaches much less importance to biospheric motives than the other groups, which is in accordance with findings of a systematic review of Holler et al. (21) that values of vegetarians are more based on universalism as opposed to omnivores.

#### Meat alternatives

Meat abstainers consume 3-component meals and combined meals without meat almost equally frequently. The products that meat abstainers use to replace meat in both types of meals are very similar, suggesting that the distinction in type of meal is less interesting than we expected (68), although the use of plant-based meat substitutes is particularly high in 3-component meals. Compared to the other two groups, meat abstainers less frequently use fish as a replacer for meat and pulses, mushrooms and nuts more frequently. Cheese and eggs are frequently used to replace meat in all groups. These findings largely agree with a study by Lehto et al. (56), based on a large-scale study in Finnish adults. The differences across the three groups are maybe most clear in their food preferences, with a much higher appreciation of plant-based protein sources among meat avoiders, a high appreciation of non-meat animal-based proteins across committed meat reducers and a high appreciation of meat products among avid meat eaters. This calls in mind Twigg’s hierarchy of foods (51), in which meat is placed at the top, providing most status, followed by animal-sourced non-meat products such as fish, eggs and cheese, and plant-based foods are placed in the bottom of the hierarchy. This hierarchy is confirmed by avid meat eaters but turns around for meat abstainers. Our findings show that the hierarchy of preferences of Dutch meat-eating adults that was found earlier (20, 69), still holds. This suggests hardly any change. We add by showing that meat abstainers have a completely different preference, as they rank plant-based protein sources at the top.

#### Meating halfway

All in all, our results show that meat abstainers are more ethically motivated than both committed meat reducers and avid meat eaters, which has also been witnessed in studies by De Backer and Hudders (27), De Backer and Hudders (28), and Rosenfeld et al. (37). Rosenfeld et al. (37) concluded that for meat abstainers, their diet is a much more central component of their self-identity. This centrality might be something that meat abstainers have in common with avid meat eaters, since heavy meat consumption is often associated with identity-aspects such as masculinity and status (70, 71).

Committed meat reducers—who consume meat 1 or 2 days a week—take position between the two poles of meat-attached and meat-abstaining consumers in terms of their attitudes and norms. Their motives are similar to meat abstainers, but the fact that they not fully abstain from meat may result in that they are perceived as more progressive than the other two groups. This reasoning concurs with a study by Patel and Buckland (72) who showed that meat reducers are perceived more positively than vegetarians and meat eaters. According to Patel and Buckland (72), social influence is strongest when groups are perceived as aspirational or positive and therefore the group of committed meat reducers may have potential to stimulate a shift toward more plant-based diets. Perhaps even more so than meat abstainers.

### Implications

#### Masculinity and morality

This study provides leads for communication, policy measures and interventions targeted at the three included subgroups, in order to stimulate a further shift toward more plant-based diets. First of all, in terms of demographic profiles it is clear that there is much more work to do in targeting males compared to females. Meat consumption is deeply associated with masculinity in western cultures (73), which might be a huge barrier toward meat reduction among males (74). Messages that counteract this stereotype of meat-eating males could possibly help to overcome this barrier. Rosenfeld and Tomiyama (57) argue that it is helpful in this respect to distinguish several types of meat (such as beef vs. chicken), as these are differently associated to traditional gender roles. In addition, meat reduction seems specifically adopted by higher educated consumers. Communications on this topic should therefore also be targeted at lower educated subgroups of the population.

Second, the difference between meat abstainers on the one hand and committed meat reducers and avid meat eaters on the other are particularly striking in terms of animal welfare issues. Animal welfare seems the discriminating motive that makes meat abstainers so dedicated in translating their attitudes or intentions into actual behavior. This accords with findings by Hopwood et al. (31), that vegetarians are more motivated by animal rights than omnivores and may be explained by aversion, or even feelings of disgust toward consuming meat (75, 76). Although committed meat reducers also value animal welfare, their opinion is less pronounced. In addition, meat abstainers and committed meat reducers and avid meat eaters differ greatly in terms of their attachment to meat. Committed meat reducers and avid meat eaters express much less feelings of repulsion. In accordance with this, they much less dislike animals as source of meat. Altogether these insights suggests that animal welfare reasons are important to focus on. Although environmental concerns are often used as a reasoning behind the aim to shift diets, and are often found as motives for meat reduction, the importance of animal welfare is not to be underestimated. Piazza (77), therefore, suggests to develop interventions that make people connect animal products with their animal origin. This engages with other recent studies advocating to target meat eaters with animal welfare messages, for example by highlighting animal suffering, that appeal to (emotions related to) animal welfare in reducing meat consumption (78–82).

#### Emo and ego

Third, the aspect of feeling-good, that we found to be the second most important motive to meat abstainers for avoiding meat, is underresearched in current literature and warrants more emphasis on unconscious, affective aspects related to meat reduction, rather than conscious deliberations (83). Carfora et al. (84), for example, found that emotional messages caused a decrease in meat consumption, but informative messages did not. The relevance of emotions in intentions to consume alternatives to meat is also confirmed in a recent study by Onwezen et al. (85), who show that positive emotions are the most relevant driver for intentions (beyond motives) to consume five types of alternative protein sources to meat.

Fourth, given the high (relative) importance of egoistic motives to meat consuming groups [confirmed by Malek and Umberger (33)], interventions that address those groups should not neglect these egoistic motives. Affordability and sensory appeal are for example important motives, and this, together with the finding that avid meat eaters dislike meatless meals, implies that tasty, affordable alternatives for meat should become more available and accessible. Additionally, these alternatives should be perceived as healthy, as health is an important motive to all groups [which is, among others, confirmed by Hopwood et al. (31), Malek and Umberger (33), Hanras et al. (86), and North et al. (36)]. This is challenging and should be a focal point for producers of meat substitutes (87), and would also benefit meat abstainers, seen the high percentage that uses plant-based meat substitutes. Another motive that is important to consider is food safety, the only motive that appears in the top three motives of all three dietary groups. Such a finding is not confirmed by related and recent studies from other countries than the Netherlands by Kemper et al. (58) or Malek and Umberger (33).

#### Health issues

In the current study, we found that only meat abstainers perceive, to some extent, positive health effects of consuming less meat. The other groups do not. And avid meat eaters are even afraid that a meatless diet would result in nutritional deficiencies. This difference between meat eaters and abstainers in terms of perceived health effects coincides with Malek and Umberger (33), but in their sample, meat reducers are more positive about meatless diets in terms of health benefits. Regardless of that, they find that weaker beliefs in nutritional adequacy of meat-free diets gives a higher chance of being a reducer as opposed to an abstainer. In both studies, the (relative) importance of health as a motive to all population subgroups is shown. A study by Mullee et al. (30) showed that about 25% of omnivores agreed that consuming vegetarian meals often is unhealthy. Kemper et al. (58) found that meat eaters are less likely to agree that plant-based diets are healthy and processed meats are unhealthy than meat reducers. All in all, these findings suggest an important role for health perceptions in the transition toward less meat-centered diets. This corresponds with Kwasny et al. (80), who recommended policy makers to inform about negative health effects of meat. Similarly, Grundy et al. (79) concluded that providing information on health consequences is promising in this respect. Future interventions could emphasize the possible health benefits of meat reduction more, and better inform the public about possible negative health effects of overconsumption of (red and processed) meat.

#### The impact of norms

Fifth, this study shows that social norms to reduce meat are low in all dietary groups. This is worrisome, as a recent review on acceptance of alternative protein sources concluded that social norms are an important driver of acceptance (83). This is in line with an Australian study, showing that social norms significantly impact attitudes to lower meat consumption (88). Additionally, a study conducted in the Netherlands showed that social norms positively predict meat curtailment behavior (59). In a study on acceptance of five types of alternative proteins, social norms even appeared to be the most relevant factor in explaining acceptance (89). And in a study among students, Schenk et al. (90) found that both injunctive and descriptive social norms (together with convenience) were the most important direct determinants of meat avoidance. This is in accordance with a modeling study by Eker et al. (91) who found social norms and self-efficacy to be the main drivers of shifting diets toward more sustainable levels. Moreover, a study by Lai et al. (92) confirmed the effect of both injunctive and descriptive social norms on meat purchases. Finally, a recent meta-review concludes that providing information on social norms appears promising to reduce the consumption of animal-based products (79). As social norms are low, and typically change slowly over time, the recent body of literature on dynamic norms may be of interest here. Dynamic norms provide information on how people’s behavior is shifting and appears to be effective in triggering behavior change (93). As many consumers indicate to have lowered their meat consumption in the past year (14), this may be used as a dynamic norm in interventions.

#### Strategies to lower meat intake

Finally, our study implies that a distinction should be made between several types of meats and several types of meat alternatives, when developing strategies to encourage meat reduction. Our study shows that the dietary groups greatly differ in their relative appreciation of different types of protein sources. In general, meat abstainers prefer plant-based protein sources over animal-based sources. For them, the bottleneck is the appreciation and consumption of cheese, which has a high environmental impact (94). Informing them on this subject and enticing them toward plant-based alternatives may be an effective route. Committed meat reducers value non-meat animal-sourced foods the most. Informing them on the sustainability impact of animal-sourced foods and targeting them with environmental and animal welfare messages could be helpful [see for a related recent study (95)]. Alternatively, these heavy flexitarians could be motivated to replace meat by fish more often as a study by Broekema et al. (94) showed that fish consumption may rise in the Netherlands. This group appreciates fish and most of them use fish to replace meat. Avid meat eaters in turn prefer meat products over other protein sources and for these meat-attached food consumers it is difficult to move away from their meat-eating habits. Therefore, for this group it might be an interesting route to stimulate a shift from red and processed meat toward white meat such as chicken, with a much lower impact on the environment and health (94)—leaving detrimental impact on animal welfare of this “meat shift” aside. Also, from the perspective of traditional gender roles, it is important to consider differences between different types of meats (57). Alternatively, this group of avid meat eaters could be targeted with a strategy that de Boer and Aiking (68) described as mixed dishes, combining proteins from animal and plant origin, the strategy “less but better,” which stands for smaller portions of animal-friendly produced meat (96) or “sustainability by stealth” [e.g., hybrid meats (97)].

### Limitations and future research

As in every study, this study comes with several limitations that provide avenues for future research. Maybe the most important limitation lays in the fact that vegetarians and vegans were identified based on their self-reported identity as being a vegetarian or a vegan. We did not ask for their meat consumption frequency, which is a missed opportunity that could have been used as a check. Malek and Umberger (33) suggest that the size of this meat-abstaining groups would probably have been smaller based on food consumption frequencies. As stated by Malek and Umberger (33), future research is needed to explain these differences and to investigate effects of different classification methods.

In addition to the previous point, we measured meat consumption in number of days a week. We have no information on portion sizes. Portion size reduction can however be an effective strategy to decrease meat consumption (98, 99), particularly among subgroups that are attached to meat or find it difficult to prepare tasty low-to-non-meat meals. Future research should therefore assess the amounts of meat consumed.

Regardless of this probability of overrated numbers of meat abstainers—that possibly are not strictly abstaining from meat or animal-based products—the number of respondents that self-identified as a vegan was rather small to analyze as a separate group (see text footnote 1). Therefore, we combined vegetarians and vegans into one group of meat abstainers. Although this is common practice (35, 100), in the context of the urgently needed shift toward more plant-based diets this could be a missed opportunity. Strict vegans do not consume any animal-based foods and therefore do not need to shift their diets in this respect, whereas vegetarians may use a lot of animal-sourced alternatives for meat (such as cheese and eggs) which is not particularly desirable with respect to environmental and animal welfare issues. Malek and Umberger (33) and Lund et al. (101) show interesting differences between vegetarians and vegans in their food choice motives and ethical and utilitarian positions. More specific, Malek and Umberger (33) show that environmental impact is second most important for vegans, but does not appear in the top five of vegetarians. It would be interesting to in-depth research this group of vegans to search for learnings that can be applied to achieve shifts in other dietary consumer groups, to research how vegetarians can be convinced to reduce their non-meat animal-based consumption and to investigate how vegans can be motivated to continue with their plant-based diet as veganism appears to be the least stable diet (34).

Related to the previous issue, future research should take a closer look at strategies to reduce the consumption of non-meat animal-based products such as dairy, eggs and fish. Our research was mainly focused on meat reduction, but a shift toward more plant-based diets entails a reduction in all types of animal-sourced foods. Meat reduction has gained a lot of attention in recent literature, but much less research has been conducted on how to reduce consumption of other animal-sourced foods. This study shows that meat is often replaced by other animal-sourced products such as fish, eggs and cheese, especially among those that do not fully abstain from eating meat. In terms of environmental impact, replacing meat with other animal-derived products is not always desirable. The CO_2_ impact of cheese is for example higher than that of chicken (94, 102). The study by Malek and Umberger (33) showed that environmental impact is relatively more important to vegans than to vegetarians [MacInnis and Hodson (18) hint at a similar difference]. This suggests that attempts to reduce the consumption of non-meat animal-based products could benefit from stressing the environmental impact of the prevailing food system, and the role of animal-based products therein.

Another limitation relates to the way we questioned food choice motives. This has been done with short expressions to measure single food choice motives. Although this method has been validated (53), it is difficult to capture how respondents interpreted the concepts when rating them. Most items are obvious, but for example the item “makes me feel good” is ambiguous. The current research surprisingly showed that this motive is the second most important motive for abstainers to have stopped consuming meat. This could possibly be explained in different ways though. It may have something to do with what is known in literature as “warm glow,” positive feelings that are elicited by doing the right thing (103). Alternatively, consumers may perceive positive effects of plant-based diets on their (physical) well-being (104), although this is an underexplored area of research (105). Further research is needed on this topic of “feeling good.”

Moreover, the results are based on self-administered questionnaires. The cross-sectional design makes it impossible to draw conclusions on causality and therefore we cannot make any statements about shifts from one dietary group to another. Recent research by Milfont et al. (34) found that the probability of shifting from a meat-rich diet toward a vegetarian or vegan diet is low and previous work on flexitarianism suggests the same (14), but more research is needed on this topic. In addition, the lack of experimental elements in this study results in findings that could help to identify leads for interventions, but research is needed on how the insights from this manuscript can be turned into effective interventions that result in, preferably long-term, changes in dietary patterns of different consumer groups.

Furthermore, the data has been collected prior to the COVID-19 outbreak. It has been found that the impact of COVID-19 on dietary patterns was modest for most of the Dutch (106), although for per-capita meat consumption some reduction was observed (13). Such outcomes give reason to expect comparable results if we would repeat this study, but future research needs to confirm this presumption.

A final limitation has to do with cross-cultural validity. This study has been conducted in the Netherlands. The Dutch diet is characterized by a large proportion of so-called 3-component meals, consisting of three separate components for proteins (mostly meat), starch and vegetables. This type of main meal is not common practice in other cultures and therefore future research is needed in other countries to confirm our findings for other cultures. With respect to meat moderation and meat avoidance in other cultures it is also interesting to address whether and how religious beliefs play an influential role in animal-based food choices. In the current work religion was not explicitly taken into account. The Netherlands is a highly-secularized country though, but in other countries and regions religious reasons may be more important. Particularly in the field of vegetarianism it is not uncommon to pay some attention to religion (18, 35).

## Conclusion

We conclude that meat abstainers differ from committed meat reducers and from avid meat eaters with respect to their socio-demographic characteristics, attitudes and norms, motives and food preferences and consumption. The results show that although differences across the groups are gradual and in the expected direction, interesting differences exist in motivations, particularly the valuation of animal welfare and “feeling good.” Moreover, in terms of valuation of protein sources the three groups are very distinct, which makes it unlikely to expect big shifts from one group to another in the short term. In view of the urgent need to move away from meat-heavy diets this is not entirely positive when it comes to expecting massive meat-reduced consumption behavior in the very near future.

## Data availability statement

The raw data supporting the conclusions of this article will be made available by the authors, without undue reservation.

## Ethics statement

Ethical review and approval was not required for the study on human participants in accordance with the local legislation and institutional requirements. Written informed consent for participation was not required for this study in accordance with the national legislation and the institutional requirements.

## Author contributions

MV: conceptualization, methodology, formal analysis, and writing—original draft, review, and editing. HD: conceptualization, methodology, and writing—original draft, review, and editing. Both authors contributed to the article and approved the submitted version.

## Footnotes

1 We fully realize that there are and could be in-group differences in vegetarians and vegans as well as differences between both meat-abstaining diet groups. However, in the present work we took both groups together because the vegans in our sample constituted a small group (*n* = 24), and their integration into one dietary category of meat abstainers is further justified by the fact that closer scrutiny revealed that both groups did not differ much except for vegans being slightly younger than vegetarians. With respect to the group of committed meat reducers or heavy flexitarians included in this study, it is relevant to point out that there is no single agreed-upon definition of flexitarianism [see further in Dagevos (10)]. A flexitarian occasionally eats meat, where it varies how often. Therefore, flexitarians could be subdivided into light, medium or moderate, and heavy flexitarians, for example, based on the number of days meat is eaten.
